# Complete genome sequence of *Allochromatium vinosum* DSM 180^T^

**DOI:** 10.4056/sigs.2335270

**Published:** 2011-12-22

**Authors:** Thomas Weissgerber, Renate Zigann, David Bruce, Yun-juan Chang, John C. Detter, Cliff Han, Loren Hauser, Cynthia D. Jeffries, Miriam Land, A. Christine Munk, Roxanne Tapia, Christiane Dahl

**Affiliations:** 1 Institute for Microbiology & Biotechnology, Rheinische Friedrich-Wilhelms-Universität Bonn, Bonn, Germany; 2 DOE Joint Genome Institute, Walnut Creek, California, USA

**Keywords:** purple sulfur bacteria, *Chromatiaceae*, versatile

## Abstract

*Allochromatium vinosum* formerly *Chromatium vinosum* is a mesophilic purple sulfur bacterium belonging to the family *Chromatiaceae* in the bacterial class *Gammaproteobacteria*. The genus *Allochromatium* contains currently five species. All members were isolated from freshwater, brackish water or marine habitats and are predominately obligate phototrophs. Here we describe the features of the organism, together with the complete genome sequence and annotation. This is the first completed genome sequence of a member of the *Chromatiaceae* within the purple sulfur bacteria thriving in globally occurring habitats. The 3,669,074 bp genome with its 3,302 protein-coding and 64 RNA genes was sequenced within the Joint Genome Institute Community Sequencing Program.

## Introduction

Anoxygenic purple sulfur bacteria are *Gammaproteobacteria* whereas chemotrophic sulfur-oxidizing bacteria are found in four classes (*Alphaproteobacteria*, *Betaproteobacteria*, *Gammaproteobacteria* and *Epsilonproteobacteria*) of the *Proteobacteria*. Strain DSM 180^T^ (= ATCC 17899 = D = NBRC 103801) is the type strain of the species *Allochromatium vinosum*, which belongs to the *Chromatiaceae*, one of currently five families in the order *Chromatiales*. Species belonging to the families *Chromatiaceae* and *Ectothiorhodospiraceae* are mainly anoxygenic photolithoautotrophic bacteria, which are able to oxidize various sulfur compounds. Anoxygenic purple sulfur bacteria like *A. vinosum* flourish wherever light reaches sulfidic water layers or sediments and often occur as dense accumulations in conspicuous blooms in freshwater as well as in marine aquatic ecosystems. Here, they are major players in the reoxidation of sulfide produced by sulfate-reducing bacteria in deeper anoxic layers. In contrast to anoxygenic purple sulfur bacteria of the family *Ectothiorhodospiraceae* and the only very distantly related green sulfur bacteria, members of the family *Chromatiaceae* like *A. vinosum* store sulfur globules inside of the cells when oxidizing sulfide or thiosulfate. They have this trait in common with a large number of environmentally important chemotrophic sulfur oxidizers like *Beggiatoa* or the sulfur-oxidizing bacterial symbionts of marine animals like *Riftia pachyptila* or *Olavius algarvensis*. Anoxygenic purple sulfur bacteria are also important primary producers of fixed carbon (up to 83% of primary production in lakes can be anoxygenic) [1]. The CO_2_ is fixed at the expense of the energy derived from the virtually unlimited and environmentally safe source of sunlight. Simultaneous with the large scale conversion of CO_2_ into organic compounds, purple sulfur bacteria oxidize reduced sulfur compounds and use these as photosynthetic electron donors [2]. In almost all freshwater and marine photic-anoxic environments, purple and also green sulfur bacteria represent the dominant anoxygenic phototrophs. Only very few and atypical ecosystems heavily polluted with organic waste have been described in which phototrophic *Alphaproteobacteria* outnumber purple sulfur bacteria. In addition to their environmental importance, purple sulfur bacteria like *A. vinosum* have a long tradition of biotechnological application not only in waste remediation and removal of toxic compounds, e.g. odorous sulfur compounds like sulfide or even explosives [3-5], but also in the production of industrially relevant organochemicals such as vitamins or biopolyesters [6-8] and the production of biohydrogen [9].

Strains of all *Allochromatium* species were isolated from freshwater, brackish water and marine habitats. *A. vinosum* is environmentally abundant and occurs not only in pelagic communities but also in littoral sediments like sandy beaches, salt marches or intertidal mud flats. Here we present a summary classification and a set of features for *A. vinosum* strain DSM 180^T^, together with the description of the complete genomic sequencing and annotation.

## Classification and features

**Figure 1:**
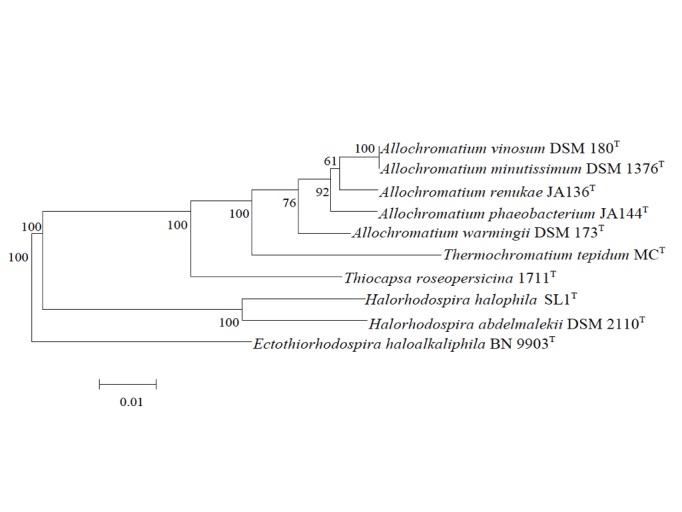
Phylogenetic tree highlighting the position of *A. vinosum* DSM 180^T^ relative to several other type strains within the *Chromatiaceae* and *Ectothiorhodospiraceae* based on 16S rRNA sequence analysis. The tree was built with the RDP Tree Builder and numbers above branches are support values from 100 bootstrap replicates [25]. Bar, 1 nucleotide substitutions per 100 nucleotides.

There are five described species currently belonging to the genus *Allochromatium* [10, Table 1] namely *A. vinosum*, *A. minutissimum,* *A. warmingii*, *A. phaeobacterium* and *A. renukae*. Figure 1 shows the phylogenetic neighborhood of *A. vinosum* DSM 180^T^ in a 16S rRNA based maximum likelihood phylogenetic tree. Based on 16S rRNA gene sequences the closest related type strain is *A. minutissimum* DSM 1376^T^ with 98.4% sequence identity, while the other type strains of the genus *Allochromatium* share 93.8-97% sequence identity.

**Table 1:** Classification and general features of *A. vinosum* DSM 180^T^ according to the MIGS recommendations [11]

**MIGS ID**	**Property**	**Term**	**Evidence code**
	Classification	Domain *Bacteria*	TAS [12]
		Phylum *Proteobacteria*	TAS [13]
		Class *Gammaproteobacteria*	TAS [14,15]
		Order *Chromatiales*	TAS [14,16]
		Family *Chromatiaceae*	TAS [17-19]
		Genus *Allochromatium*	TAS [20]
		Species *Allochromatium vinosum*	TAS [20]
		Type strain DSM 180	TAS [20]
	Gram stain	negative	TAS [20]
	Cell shape	rod	TAS [20]
	Motility	motile	TAS [20]
	Sporulation	nonsporulating	NAS
	Temperature range	25-35°C	TAS [21]
	Optimum temperature	30°C	TAS [21]
	pH range	6.5-7.6	TAS [21]
	pH optimum	7.0-7.3	TAS [21]
	Salinity	Not required but low concentrations are tolerated	TAS [21]
			
	Energy source	Photolithoautotrophic growth: H_2_, sulfide, polysulfides, thiosulfate, sulfur, sulfite Photolithoheterotrophic growth: formate, acetate, propionate, butyrate, fumarate succinate, pyruvate, malate, glycolate Chemolithoheterotrophic growth: H_2_, sulfide and thiosulfate as electron donors, acetate, propionate, pyruvate, malate, fumarate, succinate Chemolithoautotrophic growth: CO_2_ as carbon source, H_2_, sulfide, thiosulfate	TAS [21]
			
MIGS-6	Habitat	Both pelagic and in littoral sediments	TAS [21]
MIGS-15	Biotic relationship	free-living	NAS
MIGS-14	Pathogenicity	None	TAS [22]
	Biosafety level	1	TAS [22]
	Isolation site	ditch water	TAS [23]

Evidence codes - IDA: Inferred from Direct Assay (first time in publication); TAS: Traceable Author Statement (i. e. a direct report exists in the literature); NAS: Non-traceable Author Statement (i. e. not directly observed for the living, isolated sample, but based on a generally accepted property for the species, or anecdotal evidence). These evidence codes are from the Gene Ontology project [24]. If the evidence code is IDA, then the property was directly observed for a living isolate by one of the authors or an expert mentioned in the acknowledgements.

**Figure 2:**
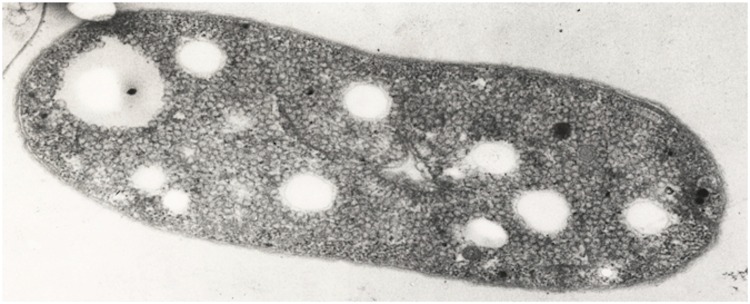
Transmission electron micrograph of a cell of *A. vinosum* strain 9011 (Photo kindly provided by Hans G. Trüper, Bonn). Magnification × 59,050. As a result of the preparation for electron microscopy, the localization of sulfur globules is visible as “holes” in the electron micrograph.

Cells of *A. vinosum* are Gram stain negative, rod shaped and about 2.0 µm x 2.5–6 µm in size [Figure 2]. There is a high intraspecies variation of the G + C content within the genus *Allochromatium*. For example the G + C content of *A. vinosum* (64.3%) and *A. warmingii* (55.1-60.2%) differs up to 10 mol % G + C content of the DNA. Cells of all species are motile and contain internal membrane structures of a vesicular type. The main carotinoid synthesized by *A. vinosum* and *A. minutissimum* belongs to the group of spirilloxanthins, whereas *A. phaeobacterium* and *A. warmingii* produce rhodopinals and *A. renukae* lycopenes, respectively. Optimal growth of *A. vinosum* is achieved within a temperature range between 25-35 °C and a pH range between 7.0-7.3, respectively [21].

Most purple sulfur bacteria grow preferentially by photolithoautotrophic oxidation of reduced sulfur compounds. However, *A. vinosum* is an ecologically significant, typically dominant inhabitant of intertidal sediments, i.e. a fluctuating environment in which redox conditions change rapidly within hours. *A. vinosum* has adapted to this environment and developed high metabolic versatility. While *A. vinosum* and *A. minutissimum* are capable of growing both photolithotrophically and chemolithotrophically the remaining species are obligate phototrophs. Photolithoautotrophic growth of *A. vinosum* occurs with hydrogen, sulfide, polysulfide, thiosulfate, sulfur and sulfite as electron donors. Light energy is used to transfer the electrons of these different compounds to the level of the more highly reducing electron carriers NAD(P)^+^ and ferredoxin for reductive carbon dioxide fixation. Photoorganoheterotrophic growth occurs with formate, acetate, propionate, butyrate, pyruvate, fumarate, succinate, malate and glycolate as organic electron donors. At reduced oxygen partial pressure even chemoorganoheterotrophic and chemolithoautotrophic growth in the dark is possible with oxygen as the terminal electron acceptor [26]. Under such conditions *A. vinosum* and *A. minutissimum* assimilate sulfate. This versatility is not shared by other anoxygenic phototrophic organisms like the green sulfur bacteria (*Chlorobiaceae*).

## Genome sequencing and annotation

### Genome project history

This organism was selected for sequencing on the basis of its environmental abundance and importance, its capability to produce hydrogen and its accessibility by manipulative genetics for biotechnology. The genome project is deposited in the Genomes OnLine Database [27] and the complete genome sequence is available in GenBank. Sequencing, finishing and annotation were performed by the DOE Joint Genome Institute (JGI). A summary of the project information is shown in Table 2.

**Table 2:** Genome sequencing project information

**MIGS ID**	**Property**	**Term**
MIGS-31	Finishing quality	Finished
MIGS-28	Libraries used	A Titanium draft library (319.2 +/- 157.8 bp insert size), two paired end (15712 +/- 3928 bp, 15,574 +/- 3,893 bp insert sizes) and one Illumina library (36 bp insert size)
		
MIGS-29	Sequencing platforms	454 Titanium, Illumina
MIGS-31.2	Sequencing coverage	136× 454 Titanium, 30× Illumina GAii
MIGS-30	Assemblers	Newbler, phrap
MIGS-32	Gene calling method	Prodigal, GenePRIMP
		
	INSDC ID	CP001896 (chromosome) CP001897 (plasmid pALVIN01) CP001898 (plasmid pALVIN02)
		
	GenBank Date of Release	August 1, 2010
	GOLD ID	Gc01210
	NCBI project ID	32547
	IMG Taxon ID	646564502
MIGS-13	Source material identifier	DSM 180
	Project relevance	Biotechnological, Environmental, Hydrogen production

### Growth conditions and DNA isolation

*A. vinosum* DSM 180^T^ was grown anaerobically in the light in RCV medium [28] at 30°C. DNA was isolated from 50 mg cell pellet by sarcosyl lysis according to the method of Bazaral and Helinski [29]. Briefly, the cell pellet was washed in Tris-EDTA buffer at pH 8, harvested and resuspended in 2 ml ice-cold TES buffer at pH 8. Cells were harvested, mixed with 250 µl sucrose solution (20% (w/v) sucrose in TES) and incubated for 30 min on ice. Afterwards, 250 µl of lysozyme RNAse solution (20 mg/ ml lysozyme, 1 mg/ ml RNAse) were added followed by a further incubation for 30 min at 37 °C with gentle shaking. 100 µl sarcosine solution (10% (w/v) laurylsarcosine, 250 mM EDTA) were added and the sample was pressed through a sterile cannula (1.2 × 49 mm). DNA purification was carried out by phenol/ chloroform extraction. Finally, purified DNA was transferred into Tris-EDTA buffer at pH 8 by dialysis. The quality and quantity of extracted DNA was evaluated using the DNA Mass Standard Kit provided by the JGI.

### Genome sequencing and assembly

The genome of *Allochromatium vinosum* DSM 180 was sequenced at the Joint Genome Institute (JGI) using a combination of Illumina [30] and 454 technologies [31]. An Illumina GAii shotgun library with reads of 36 bp, a 454 Titanium draft library with average read length of 319.2 +/- 157.8 bp bases, and 2 paired end 454 libraries with average insert sizes of 15712 +/- 3928 bp and 15574 +/- 3,893 bp were generated for this genome. All general aspects of library construction and sequencing performed at the JGI can be found at the JGI website [32]. Illumina sequencing data was assembled with VELVET [33], and the consensus sequences were shredded into 1.5 kb overlapped fake reads and assembled together with the 454 data. Draft assemblies were based on 238 Mb 454 draft data and approximately 48,000 per Mb paired end data. Newbler parameters are - consed - a 50 -l 350 -g -mi 96 -ml 96 -o P_miml96_QD. The initial Newbler assembly contained 64 contigs in 41 scaffolds. The initial 454 assembly was converted into a phrap assembly by making fake reads from the consensus, collecting the read pairs in the 454 paired end library. The Phred/Phrap/Consed software package [34] was used for sequence assembly and quality assessment [35-37] in the following finishing process. After the shotgun stage, reads were assembled with parallel phrap (High Performance Software, LLC). Possible mis-assemblies were corrected with gapResolution (Cliff Han, unpublished), Dupfinisher [38], or sequencing cloned bridging PCR fragments with subcloning or transposon bombing (Epicentre Biotechnologies, Madison, WI). Gaps between contigs were closed by editing in Consed, by PCR and by Bubble PCR primer walks. A total of 174 additional PCR reactions were necessary to close gaps and to raise the quality of the finished sequence.

### Genome annotation

Genes were identified using Prodigal [39] as part of the Oak Ridge National Laboratory genome annotation pipeline, followed by a round of manual curation using the JGI GenePRIMP pipeline [40]. The predicted CDSs were translated and used to search the National Center for Biotechnology Information (NCBI) nonredundant database, UniProt, TIGRFam, Pfam, PRIAM, KEGG, COG, and InterPro databases. These data sources were combined to assert a product description for each predicted protein. Non-coding genes and miscellaneous features were predicted using tRNAscan-SE [41], RNAMMer [42], Rfam [43], TMHMM [44], and SignalP [45].

## Genome properties

**Figure 3:**
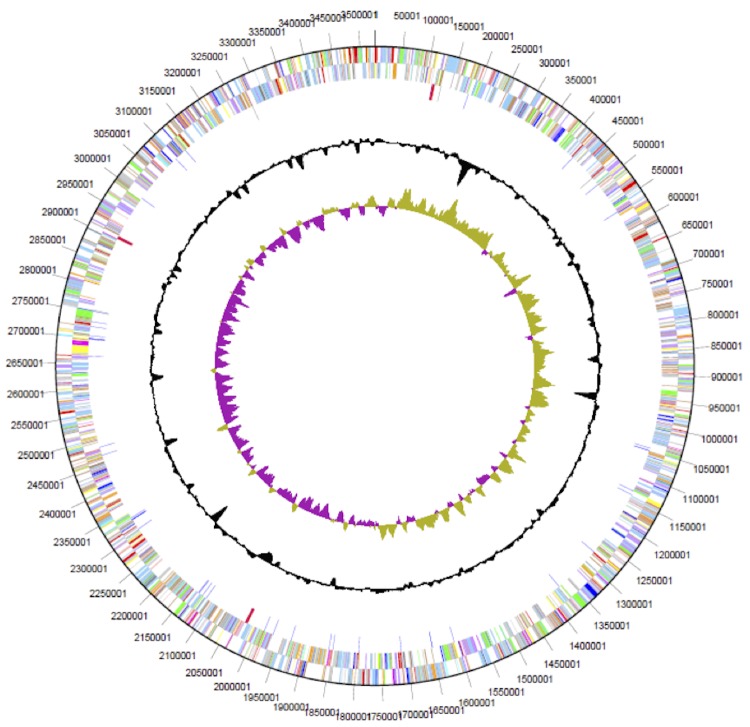
Graphical circular map of the chromosome. From outside to the center: Genes on forward strand (color by COG categories), Genes on reverse strand (color by COG categories), RNA genes (tRNAs green, rRNAs red, other RNAs black), GC content, GC skew.

**Figure 4:**
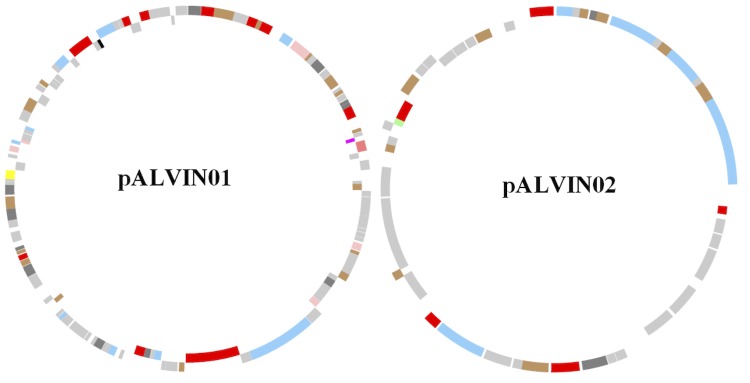
Graphical circular map of the 102.2 kbp plasmid pALVIN01 (127 genes including 8 pseudogenes) and the 39.9 kbp plasmid pALVIN02 (49 genes including 1 pseudogene). From outside to the center: Genes on forward strand (color by COG categories), Genes on reverse strand (color by COG categories), RNA genes (tRNAs green, rRNAs red, other RNAs black), GC content, GC skew.

The 3,669,074 bp genome consists of a 3,526,903 bp chromosome and two plasmids of 102,242 bp and 39,929 bp, respectively. The genome exhibits an overall G + C content of 64.19% (Table 3, Figure 3, and Figure 4). Of the 3,366 genes predicted, 3,302 are protein-coding genes and 64 RNAs; 82 pseudogenes were also identified. The majority of the protein-coding genes (73.26%) were assigned a putative function while the remaining ones were annotated as hypothetical proteins. The distribution of genes into COGs functional categories is presented in Table 4.

**Table 3:** Genome Statistics

**Attribute**	**Value**	**% of Total**
Genome size (bp)	3,669,074	100.00%
DNA coding region (bp)	3,324,920	90,62%
DNA G+C content (bp)	2,355,037	64.19%
Number of replicons	3	
Extrachromosomal elements	2	
Total genes	3,366	100.00%
RNA genes	64	1.90%
rRNA operons	3	
Protein-coding genes	3,302	98.10%
Pseudo genes	82	2.44%
Genes with function prediction	2,466	73.26%
Genes in paralog clusters	413	12.27%
Genes assigned to COGs	2,505	74.42%
Genes assigned Pfam domains	2,662	79.08%
Genes with signal peptides	649	19.28%
Genes with transmembrane helices	771	22.91%
CRISPR repeats	3	

**Table 4:** Number of genes associated with the general COG functional categories

**Code**	**Value**	**%age**	**Description**
J	160	5.70	Translation, ribosomal structure and biogenesis
A	1	0.04	RNA processing and modification
K	106	3.78	Transcription
L	145	5.17	Replication, recombination and repair
B	1	0.04	Chromatin structure and dynamics
D	51	1.82	Cell cycle control, cell division, chromosome partitioning
Y	0	0.00	Nuclear structure
V	49	1.75	Defense mechanisms
T	303	10.80	Signal transduction mechanisms
M	198	7.06	Cell wall/membrane biogenesis
N	1,135	4.81	Cell motility
Z	0	0.0	Cytoskeleton
W	0	0.0	Extracellular structures
U	84	2.99	Intracellular trafficking and secretion
O	126	4.49	Posttranslational modification, protein turnover, chaperones
C	224	7.98	Energy production and conversion
G	101	3.60	Carbohydrate transport and metabolism
E	148	5.27	Amino acid transport and metabolism
F	54	1.92	Nucleotide transport and metabolism
H	159	5.67	Coenzyme transport and metabolism
I	63	2.25	Lipid transport and metabolism
P	172	6.13	Inorganic ion transport and metabolism
Q	41	1.46	Secondary metabolites biosynthesis, transport and catabolism
R	261	9.30	General function prediction only
S	224	7.98	Function unknown
-	861	25.58	Not in COGs

## Insights from the genome sequence

### Extrachromosomal elements

*A. vinosum* contains two extrachromosomal elements of 102,242 bp (pALVIN01) and 39,929 bp (pALVIN02), respectively, in accordance with previous analyses performed in our laboratory [46]. A third plasmid claimed by Gaju *et al*. 1995 is not present [47]. None of the genes identified on the plasmid encode for proteins of central metabolic pathways. It is noteworthy that plasmid pALVIN02 has a low GC content of only 53.5% as compared to the 64.26% and 61.9% of the main chromosome and pALVIN01, respectively.

### Phototrophy

*A. vinosum* employs type II reaction centers to convert light energy into electrochemical energy. Three subunits of the photosynthetic reaction center, *pufC*, *pufM* and *pufL* are clustered and co-transcribed together with three sets of *pufA* and *pufB* genes encoding for light-harvesting complex (LH1) apoproteins (Alvin_2547-2555) [48]. Subunit four of the reaction center, *pufH*, is located upstream, next to two genes encoding for photosynthetic complex assembly proteins and a hypothetical protein that probably constitutes an additional complex assembly protein (Alvin_2634-2637). Blast searches indicate that the genome contains six individual sets encoding for LH2 apoproteins, Alvin_0703-0706 and Alvin_0708-0709, which constitute one cluster together with *lux* genes, Alvin_2576-2579 and Alvin_2759-Alvin_2760. High copy numbers of different light harvesting subunits might allow adapting to alternating growth conditions such as light intensity or temperature [49]. The main carotenoids synthesized by *A. vinosum* belong to the group of spirilloxanthins. Genes necessary for spirilloxanthin biosynthesis starting from the C-5 compounds dimethylallyl-PP and isopentyl-PP are located next to each other from Alvin_2564 to Alvin_2570. *A. vinosum* utilizes only one type of bacteriochlorophyll (BChl), namely BChla. Genes necessary for conversion of the heme biosynthesis intermediate protoporphyrin IX into Bchla are partly distributed over the genome (Alvin_1182-1183, 2224, 2556, 2561-2563, 2632, 2638-2643 and 2645-2646).

### Diffusible electron carriers

During photosynthesis, diffusible electron carriers maintain light-driven cyclic electron flow by channeling electrons from the cytochrome bc_1_ complex back to the reaction center. In most cases this feature is mediated by mobile, soluble electron-carrying proteins located in the periplasm. In non-sulfur purple bacteria, the high potential cytochrome c_2_ represents the main diffusible electron carrier. However, in *A. vinosum* cytochrome c_2_ function is replaced by other high potential c-type cytochromes and a high potential iron sulfur protein (HiPIP) [50]. HiPIP is encoded by Alvin_2274 with an N-terminal signal peptide for periplasmic translocation [51] and is able to act as direct reductant for the reaction center in *A. vinosum* [52,53] and other *Chromatiaceae* [54-56]. It occurs in high concentrations and is favored over high potential c-type cytochromes under photolithoautotrophic conditions [53]. HiPIP was also shown to operate as an acceptor of electrons released by thiosulfate dehydrogenase during oxidation of thiosulfate [57]. Besides HiPIP, the genome of *A. vinosum* reveals the genetic information for several high potential c-type cytochromes. Alvin_1694 encodes for cytochrome c_8_ (c_551_) that is involved in transferring electrons to the photosynthetic reaction center [58]. It appears in lower amounts than HiPIP but the ratio of HiPIP to cytochrome c_8_ is slightly higher under autotrophic conditions with Na_2_S and Na_2_S_2_O_3_ than in presence of organic compounds [53]. Similar to *Rubrivivax gelatinosus* [59] in *A. vinosum* appears at least one cytochrome c_8_ isoenzyme, namely Alvin_1093 encodes for a cytochrome that is part of flavocytochrome c. The protein expressed by Alvin_2879 reveals high similarities to the flavocytochome c diheme subunit [60] and its amino acid sequence is nearly identical to recently discovered cytochrome c_4_ of *Thiocapsa roseopersicina* [61]. It is reported to appear both in a soluble and a membrane-bound form in *A. vinosum* [62]. Cytochrome c’ is another high potential c-type cytochrome encoded by Alvin_2765 [63] with no clearly determined function up to date. It was postulated to take part in electron transfer [64] but also to bind NO [65] in order to provide resistance to nitric oxide [66]. Besides these already well described cytochromes the genome reveals three more potential diffusible c-type cytochromes, namely Alvin_0020, Alvin_0023 and Alvin_1846. Alvin_0020 and Alvin_0023 are annotated as soluble diheme c-type cytochromes and are localized in adjacent gene clusters of unknown function. Alvin_1846 is annotated as a further isoenzyme of cytochrome c_8_ and blast searches reveal most similarities to a cytochrome c_553_ from *Magnetospirillum gryphiswaldense* MSR-1.

### Autotrophic growth with carbon dioxide

In a wide range of autotrophic organisms, the ability to grow autotrophically with carbon dioxide as the sole carbon source is due to the enzyme ribulose 1,5-biphosphate carboxylase/oxygenase (RuBis-CO). *A. vinosum* possesses two complete sets of genes encoding for large and small RuBis-CO subunits namely RbcA/ RbcB and RbcS/ RbcL represented by Alvin_1365/1366 and Alvin_2749/2750, respectively [67]. RuBis-CO of RbcAB is the major species present in *A. vinosum* under standard photolithoautotrophic growth conditions [68]. According to the gene arrangement, RuBis-CO genes *rbcAB* belong to form IAq RuBis-CO genes that are typically associated with *cbbQ* genes that encode for proteins affecting activity of RuBis-CO [69]. Upstream, a gene encoding for a member of the LysR family of transcriptional regulators might regulate expression of *rbcAB* [70]. RuBis-CO genes *rbcSL* represent form IAc RuBis-CO genes that are always associated together with a cluster of α-carboxysome genes [71]. Expression studies in *Hydrogenovibrio marinus* revealed a preferential expression of RuBis-CO form IAc at low CO_2_ concentrations, whereas expression of form IAq predominates at high CO_2_ concentrations. Thus RuBis-CO RbcSL in co-occurrence with carboxysomes might allow *A. vinosum* to grow at very low CO_2_ concentration. But so far carboxysomes have never been reported for *A. vinosum*. Interestingly, downstream of *rbcL* a second gene for a small subunit of RuBis-CO is found but its role during carbon dioxide fixation still has yet to be determined. Besides the RuBis-CO genes dedicated to carbon fixation, *A. vinosum* harbors a gene (Alvin_2545) encoding for a form IV RuBis-CO-like protein (RLP). These types of RuBis-CO enzymes do not catalyze ribulose 1,5-bisphosphate dependent CO_2_ fixation but might be involved in sulfur metabolism and stress response [72,73] as well as in methionine salvage pathway [71]. The role of the RLP in *A. vinosum* is still not resolved.

### Dissimilatory sulfur metabolism

The capability to oxidize reduced sulfur compounds is the central feature of *A. vinosum* during photolithoautotrophic growth. Light energy is used to transfer electrons from reduced sulfur compounds to the level of the more highly reducing electron carriers NAD(P)^+^ and ferredoxin for reductive carbon dioxide fixation. The most abundant reduced sulfur component in the habitat of *A. vinosum* is sulfide but up to the time of publication of this report, it is still not clear which enzymes are responsible for sulfide oxidation in *A. vinosum*. One possibility is the oxidation via a soluble periplasmic flavocytochrome c. The protein is a heterodimer consisting of a 21 kDa di-heme cytochrome c subunit (FccA) and a 46 kDa flavin-binding subunit (FccB) [2]. Both subunits are represented in the genome of *A. vinosum* by Alvin_1093 and Alvin_1092, respectively. Flavocytochrome c catalyzes the oxidation of sulfide to sulfur or polysulfides *in vitro* using soluble c-type cytochromes as electron acceptors [74] but the exact role of this protein *in vivo* is still not resolved. Mutants in which the genes *fccAB* are inactivated by a kanamycin cassette oxidize sulfide with rates similar to the wild type [74]. Some sulfide-utilizing green sulfur bacteria, e.g. *Chlorobium luteolum* (formerly *Pelodictyon luteolum*), and purple sulfur bacteria, e.g. *Thiocapsa roseopersicina*, *Thiococcus pfennigii* (formerly *Thiocapsa pfennigii*) and *Allochromatium warmingii* (formerly *Chromatium warmingii*), do not produce flavocytochrome c, which is an additional hint that flavocytochrome c is not essential for sulfide oxidation [2].

Other candidate proteins for oxidizing sulfide are sulfide:quinone oxidoreductases (SQRs). This type of enzyme catalyzes the oxidation of sulfide with an isoprenoid quinone as electron acceptor. SQR activity was shown to occur biochemically in membrane-bound form in *A. vinosum* [74]. Polysulfides were identified as main reaction products *in vitro* for *Rhodobacter capsulatus* [75] that might further react chemically [76] or enzymatically [77] to sulfur for storage in sulfur globules. Sequence data reveal two genes encoding for SQRs in *A. vinosum* (Alvin_1195 and Alvin_2145). Alvin_2145 represents a SQR of type IV [78], of which SqrD, an orthologous enzyme, is responsible for most of the SQR activity in the green sulfur bacterium *Chlorobaculum tepidum* [79,80]. A correlation between the occurrence of SqrD and the production of intracellular sulfur globules has been noted: *sqrD* genes are present in members of the *Chromatiaceae* (e.g. *Thiococcus pfennigii*, *Thioflavicoccus mobilis* and *Marichromatium purpuratum*) while they are absent in those species of the family *Ectothiorhodospiraceae* that produce exclusively extracellular sulfur globules [78]. The enzyme encoded by Alvin_1195 belongs to type VI SQRs. The orthologous enzyme SqrF of *C. tepidum* is important for growth at high sulfide concentrations [78].

The *sox* gene cluster of the chemolithoautotrophic Alphaproteobacterium *Paracoccus pantotrophus* comprises 15 genes that encode proteins involved in the oxidation of thiosulfate, whereas the protein products of 7 genes *soxXYZABCD* are sufficient for oxidizing thiosulfate *in vitro* [81,82]. In *A. vinosum*, *sox* genes are separated into three gene clusters. Cluster one comprises Alvin_2108 to Alvin_2112. However, only Alvin_2011 and Alvin_2012 encode for Sox proteins, namely SoxY and SoxZ. The second gene cluster extends between Alvin_2165 and Alvin_2167 with Alvin_2167 encoding for SoxB. The third gene cluster includes Alvin_2168 to Alvin_2182, where SoxX, SoxA, SoxK and SoxL are encoded by Alvin_2168 to Alvin_2171. The additional genes of cluster three mainly encode for cytochrome c biogenesis proteins. The Sox protein complex is localized in the periplasm. In *A. vinosum* SoxCD activity of *P. pantotrophus* probably is replaced by sulfur transferase activity of SoxL transferring the sulfane atom for storage to sulfur globules, thus regenerating the cysteine residue of SoxY [83]. SoxK acts as binding protein keeping SoxX and SoxA together.

Under slightly acidic conditions thiosulfate is increasingly oxidized to tetrathionate via thiosulfate dehydrogenase encoded by Alvin_0091 [84]. This periplasmic enzyme is a c-type cytochrome and acts as monomer [84].

Rhodaneses (thiosulfate sulfurtransferases) are enzymes that catalyze the transfer of the sulfane sulfur atom of thiosulfate to cyanide producing thiocyanate (SCN^-^) and SO_3_^2-^ *in vitro*. In addition to SoxL, six other genes are annotated that might encode rhodaneses, namely Alvin_0258, Alvin_0866, Alvin_0868, Alvin_1587, Alvin_2599 and Alvin_3028. While Alvin_0258 encodes a signal peptide for periplasmic localization Alvin_3028 is proposed to encode a transmembrane protein. The remaining proteins lack both a signal peptide and transmembrane domains and are therefore expected to be cytoplasmic. Roles for these proteins in dissimilatory sulfur metabolism are currently unclear.

Uptake and oxidation of external, insoluble elemental sulfur by *A. vinosum* and other purple sulfur bacteria as well as by green sulfur bacteria is still unresolved and the *A. vinosum* genome does not reveal any new insights.

Oxidation of sulfide, thiosulfate and sulfur leads to the formation of sulfur globules in the periplasm for storage of still oxidizeable sulfur compounds. The envelope of these globules consists of three constitutively synthesized hydrophobic proteins, namely SgpA, SgpB and SgpC that are encoded by Alvin_1905, Alvin_0358 and Alvin_1325, respectively. SgpC plays an important role in globule expansion, whereas SgpA and SgpB can be replaced by each other to some extent [85,86]. Sulfur inside these globules is present as mono- and bis-organyl sulfanes [87]. For further oxidation of stored sulfur globules sulfur probably is reductively activated and transported into the cytoplasm via a perthiolic carrier molecule. For oxidation of stored sulfur the dissimilatory sulfite reductase (Dsr), proteins are of essential importance since several *A. Vinosum* dsr mutants are unable to oxidize stored sulfur. Dsr proteins are encoded by a single *dsrABEFHCMKLJOPNRS* gene cluster extending from Alvin_1251 to Alvin_1265. Transferred sulfur could be released in the cytoplasm from the perthiolic organic carrier molecule as sulfide and subsequently transferred to dissimilatory sulfite reductase DsrAB via sulfur relay system involving the proteins DsrC and DsrEFH. DsrAB is homologous to sulfite reductase of sulfate reducing organisms [88] and therefore might operate in the opposite direction in sulfur-reducing prokaryotes [89,90]. Electrons released from the oxidation might be fed into the photosynthetic electron transport via the transmembrane DsrMKJOP complex [91]. DsrN probably catalyzes the glutamine dependent amidation of siroheme, a cofactor of DsrAB. The function of DsrL, DsrR and DsrS is still not resolved but DsrL appears to play an essential role during oxidation of stored sulfur since *dsrL* in-frame mutants are unable to oxidize stored sulfur [92-97]. While the *dsr* genes are transcribed as one single element, *dsrC* and *dsrS* each have an additional independent promoter site [98].

Besides *dsrC,* there are four more genes annotated as TusE/DsrC/DsvC family sulfur relay proteins, namely Alvin_0028, Alvin_0345, Alvin_0732 and Alvin_1508. Whether the encoded proteins are involved in dissimilatory sulfur metabolism and/ or thiouridine biosynthesis as reported for TusE in *Escherichia coli* [99] and possibly in other cellular processes remains to be established.

Detection of all necessary enzymes for oxidation of sulfite to sulfate has still not been achieved. Indirect oxidation of sulfite to sulfate can take place via APS reductase and ATP sulfurylase. Both enzymes are encoded in *A. vinosum* by Alvin_1119 to Alvin_1121 and Alvin_1118, respectively. APS reductase is membrane-bound via AprM encoded by Alvin_1119. Both APS reductase and ATP sulfurylase seem to have only a minor influence on sulfite oxidation in *A. vinosum* since mutants with inactivated *aprAB* genes reveal sulfite oxidation rates similar to the wild type [100]. Other interesting genes encoding for proteins probably involved in dissimilatory sulfur metabolism are represented by Alvin_1317 to Alvin_1319. These genes encode for sulfur reductase subunits annotated as SreA, SreB and SreC with the ability also to reduce polysulfide in *Archaea*. Subunit SreA is predicted to occur in the periplasm. Blast searches reveal high similarities to corresponding genes in *Acidithiobacillus ferrooxidans* and other sulfur oxidizing *Bacteria* and *Archaea* [101] but the specific function in *A. vinosum* still has to be determined.

### Heterotrophic growth with organic substrates

With respect to utilizable substrates as electron donators for photosynthesis, *A. vinosum* is very versatile. Besides autotrophic growth with reduced sulfur compounds, *A. vinosum* is able to assimilate a great number of organic substrates during photoorganoheterotrophic growth [21]. Fumarate, succinate, malate and pyruvate are oxidized by a complete tricarboxylic acid cycle. Similarly, acetate is utilized, converting it to acetyl-CoA via acetate-CoA-ligase (Alvin_0165) or the successive reactions of acetate kinase (Alvin_0610) and phosphate acetyltransferase (Alvin_0609). Propionate might be channeled into tricarboxylic acid cycle for oxidation at the stage of succinyl-CoA, but the enzyme responsible for converting 2-methylcitrate to 3-hydroxybutane-1,2,3-tricarboxylate is still not detected. Formate and glycolate are utilized as electron donators via formate dehydrogenase (Alvin_2451-2454) and glycolate dehydrogenase (Alvin_0157-0158, 0174 and 1931), respectively. Malate synthase G (Alvin_2606) catalyzes the formation of malate from glyoxylate, which can then enter the tricarboxylic acid cycle. Heterotrophic growth with butyrate is reported as variable [21]. This observation is consistent with the fact that the genome sequence reveals no obvious genes encoding proteins for butyrate oxidation. Although *A. vinosum* possess the complete enzymatic facility for glycolysis, this organism is not able to grow with glucose as an electron donor. This incapability is probably due to the lack of a phosphotransferase system for glucose uptake.

### Chemotrophy

Besides phototrophic growth, *A. vinosum* is also able to grow chemotrophically with oxygen as an electron acceptor under low oxygen concentration [26]. The genome sequence of *A. vinosum* reveals the presence of two oxidases working preferentially under microaerobic conditions. Alvin_2499 and Alvin_2500 encode for a cytochrome *bd* oxidase that is widely distributed among *Archaea* and *Bacteria* [102]. The function of the *bd* oxidase is to scavenge toxic oxygen during nitrogen fixation as it was shown to be co-transcribed with a nitrogen fixation related gene that is located upstream of oxidase subunit I [26]. Furthermore, *A. vinosum* possesses a cytochrome *cbb_3_* oxidase (Alvin_0781 to Alvin_0784), a member of the heme-copper oxidase family. Cytochrome *cbb_3_* oxidase is characterized by its ability to maintain catalytic activity under low oxygen concentration and the capability to translocate protons. The genomic arrangement corresponds to the well characterized *cbb_3_* oxidase of *Rhodobacter capsulatus* [103].

### Hydrogenases

Hydrogenases are oxidoreductases with the ability either to oxidize hydrogen as energy source or to produce hydrogen as an electron sink. According to their metal content, hydrogenases are divided into NiFe, FeFe and iron sulfur free hydrogenase classes. The genome of *A. vinosum* reveals the presence of four individual NiFe hydrogenases. The typical protein structure of NiFe hydrogenases is heterodimeric and the correct folding, maturation and incorporation of ligands require accessory proteins. The membrane bound hydrogenase of *A. vinosum* DSM 185 encoded by Alvin_2036 and Alvin_2039 in strain DSM 180^T^ is well characterized [104]. The structural genes *hydS* and *hydL* are separated by an intergenic sequence encoding a membrane-bound b-type cytochrome and a soluble iron-sulfur protein, respectively, with similarities to DsrM and DsrK [105,106]. Genomic organization corresponds to *hynSL* (former *hydSL*) in the photosynthetic bacterium *Thiocapsa roseopersicina* BBS, also belonging to the family *Chromatiaceae* [107]. Hyn hydrogenase is membrane-bound facing the periplasmic side, constitutively expressed, and physiologically connected to cellular redox processes via the proteins encoded by the intergenic sequence [108,109]. Besides HydSL, *A. vinosum* encodes for a second membrane associated hydrogenase that is highly similar to *T. roseopersicina* HupSLC. In *A. vinosum*, *hupC* (Alvin_2307) encodes for a b-type cytochrome, *hupL* (Alvin_2308) and *hupS* (Alvin_2309) for large and small hydrogenase subunits, respectively. While the accessory *hupDHI* genes are required for hydrogenase biosynthesis in *T. roseopersicina* [110], in *A. vinosum* only *hupD* (Alvin_2306) is available. Hup hydrogenase of *T. roseopersicina* is also membrane -bound facing the periplasm, inducible and is involved in the oxidation of H_2_ occurring in the environment or resulting from nitrogenase activity. The b-type cytochrome represented by HupC participates in channeling electrons into the quinone pool [108,109]. High sequence similarities between *T. roseopersicina* and *A. vinosum* Hup hydrogenases might result in at least a similar localization and function. A soluble hydrogenase was recently characterized in *A. vinosum* DSM 185 [111] belonging to the HoxEFUYH type of [NiFe] hydrogenases. It consists of a hydrogenase HoxYH and a diaphorase HoxFU containing binding sites for flavin mononucleotide and NAD(H) in subunit HoxF. HoxE is the fifth subunit and is suggested to play a role in electron transfer as this was reported for the homologous hydrogenase complex in *T. roseopersicina* [112,113]. The HoxEFUYH type of hydrogenases is evolutionarily most closely related to complex I [111]. In *A. vinosum* DSM 180^T^, Hox hydrogenase is encoded by the genes Alvin_1864 to Alvin_1868 followed by a gene (Alvin_1869) encoding for a hydrogenase maturation protein, namely HoxW. In *T. roseopersicina*, HoxEFUYH seems to be connected to sulfur metabolism and dark and photofermentative processes [109]. A fourth NiFe hydrogenase is encoded by Alvin_0807 to Alvin_0810. Blast searches reveal similarities to hydrogenase/sulfur reductase of *Thermodesulfovibrio yellowstonii* DSM 11347 and *Chlorobium tepidum*, but the exact function and localization still has to be determined. Besides hydrogenase gene clusters, there are several genes annotated for hydrogenase maturation distributed all over the genome such as Alvin_0054, Alvin_1451, Alvin_1958, Alvin_2766 and Alvin_2797 to Alvin_2799. *A. vinosum* is able to use hydrogen both for phototrophic and chemotrophic growth [21], but the role of the various hydrogenases in these processes still has to be determined.

### Nitrogen metabolism

*A. vinosum* belongs to the diazotrophic bacteria encoding for iron-molybdenum dinitrogenase (*nifDK*) and dinitrogenase reductase (*nifH*). Genes involved in nitrogen fixation are spread all over the genome but always aggregated in gene clusters. The genome reveals that regulation takes place at least on two levels. Level one comprises the Ntr system consisting of the two component system NtrB/ NtrC (Alvin_2998/ Alvin_2997), nitrogen regulatory protein P-II (Alvin _0793) and the bifunctional uridylyltransferase/ UMP-removing enzyme of P-II GlnD (Alvin_2042), that is located in the same gene cluster as the membrane-bound hydrogenase HydSL and might reflect a role of this hydrogenase in metabolizing H_2_, that is released during nitrogen fixation. The NtrB/ NtrC system regulates transcription of the NifA protein (Alvin_1291) required for transcription of all *nif* genes [114]. Usually post-translational control of NifA occurs on a second level by oxygen. Therefore NifA contains either conserved cysteine residues for direct interaction with oxygen as it has been reported for *Bradyrhizobium japonicum* [115] or NifA is inhibited by NifL after oxygen did interact with this protein [116]. Neither strategy is present in *A. vinosum*, which is in agreement with observations made for *Halorhodospira halophila* [117]. Perhaps the lack of oxygen sensing ability is compensated by direct post-translational modification of nitrogenase on level three. Reversible ADP-ribosylation of dinitrogenase reductase is mediated by dinitrogenase reductase ADP-ribosyl transferase (Alvin_1882) and dinitrogenase reductase activating glycohydrolase (Alvin_3044) in response to various exogenous stimuli [118,119]. Interestingly, just as in *H. halophila* the *A. vinosum* genome reveals three distinct *rnf* gene clusters (Alvin_0562 to Alvin_0579, Alvin_1169 to Alvin_1179 and Alvin_2673 to Alvin_2681). The Rnf complex was shown to play a role in nitrogen fixation as overproduction of the *rnf* operon increased nitrogenase activity in *Rhodobacter capsulatus* [120]. Furthermore, this complex connects the cellular ferredoxin to the pyridine nucleotide pool and was also shown to generate a sodium ion gradient across the cytoplasmic membrane in *Acetobacterium woodii* [121]. The role of Rnf complexes in *A. vinosum* is entirely unclear at this point in time. Gene arrangement in cluster one with a gene encoding for a sodium/ hydrogen exchanger (Alvin_0576) for example does not exclude a function similar to that in *A. woodii* in generating a sodium gradient across the membrane. There are no obvious genes present in *A. vinosum* mediating dissimilatory or assimilatory nitrate reduction despite a single gene encoding for nitric oxide reductase subunit B (Alvin_0931) and the two component regulatory system NarX and NarL (Alvin_1057 and Alvin_1058) for sensing nitrite and nitrate.

### Assimilatory sulfur metabolism

Assimilatory sulfate reduction occurs in a variety of phototrophic purple bacteria. In contrast to more specialized species within the *Chromatiaceae* and *Ectothiorhodospiraceae*, *A. vinosum* is able to grow photoorganoheterotrophically and therefore is able to assimilate sulfate as sulfur source. Assimilatory sulfate reduction starts with the uptake of sulfate from the environment by an ABC transporter encoded by Alvin_2443 to Alvin_2441 and annotated as *cysTWA* [122]. Downstream, on the opposite strand, gene Alvin_2444 encodes for a periplasmic sulfate binding protein (CysP) mediating sulfate to the transport system. In the cytoplasm, sulfate is activated by the formation of a phosphate-sulfate anhydride bond resulting in adenosine 5'-phosphosulfate (APS). This reaction is catalyzed by the assimilatory heterodimeric ATP-sulfurylase encoded by Alvin_2448 and Alvin_2449 and annotated as *cysDN* [123]. APS is either directly reduced to sulfide by assimilatory APS reductase (*cysH*) and sulfite reductase (*cysI*) (Alvin_2447 and Alvin_2446) for incorporation in cysteine for further utilization or shuttled into a second pathway. Here, APS is initially phosporylated to phosphoadenosine phosphosulfate (PAPS) via APS kinase (CysC) encoded by Alvin_1127 [123].
